# Amygdala subregional atrophy across ATN-defined Mild Cognitive Impairment subgroups

**DOI:** 10.3389/fnins.2026.1791269

**Published:** 2026-05-18

**Authors:** Qianqian Yuan, Chen Xue, Wenzhang Qi, Xuhong Liang, Yue Tang, Yiming Ruan, Chaoyong Xiao

**Affiliations:** 1 Department of Radiology, Nanjing Chest Hospital, Affiliated Nanjing Brain Hospital, Nanjing Medical University, Nanjing, Jiangsu, China; 2 Department of Radiology, The Affiliated Brain Hospital of Nanjing Medical University, Nanjing, China; 3 Department of Radiology, Sichuan Second Hospital of Traditional Chinese Medicine, Chengdu, Sichuan, China; 4 Department of Radiology, the Affiliated Yixing Traditional Chinese Medicine Hospital of Yangzhou University, Wuxi, Jiangsu, China

**Keywords:** Alzheimer’s disease, amygdala subnuclei, ATN, Mild Cognitive Impairment, structural magnetic resonance imaging

## Abstract

**Background:**

Alzheimer’s disease (AD) pathology begins years before clinical symptoms, with Mild Cognitive Impairment (MCI) as a prodromal stage. The ATN framework (Amyloid, Tau, Neurodegeneration) aids in stratifying MCI risk. While amygdala atrophy is a recognized biomarker, amygdala subregional changes across ATN-defined MCI subgroups remain underexplored.

**Methods:**

This study analyzed MRI data and cerebrospinal fluid biomarkers from 134 MCI participants classified into A−T−, A+T−, and A+T+ subgroups. The volumes of amygdala subregions were computed and compared among the different groups. Furthermore, we also investigated the relationship between the altered brain regions and cognitive function.

**Results:**

Significant atrophy was observed in the A+T+ group within bilateral basal, accessory basal, central nuclei, and right cortical-amygdaloid transition area compared to other groups. Volume reductions in the left central nucleus correlated positively with cognitive scores.

**Conclusion:**

Amygdala subregional atrophy, particularly in the central, basal, accessory basal, and cortical-amygdaloid transition nuclei, is linked to AD pathology progression and cognitive decline. The findings suggest a potential vulnerability of the right amygdala and suggest these subregions may be associated with AD-related pathological progression.

## 1 Introduction

Alzheimer’s disease (AD) is the most common neurodegenerative disorder, characterized by progressive memory decline and multiple cognitive impairments (Scheltens et al., 2021). Its hallmark pathological features include β-amyloid (Aβ) deposition, hyperphosphorylation of tau protein, and consequent neurodegeneration and brain atrophy (Zhang J. et al., 2024). Notably, these pathological changes begin 10–20 years prior to the onset of clinical symptoms, highlighting the critical importance of early identification and intervention in high-risk individuals (Breijyeh and Karaman, 2020). Mild Cognitive Impairment (MCI) is widely considered a prodromal stage of AD, positioned between normal aging and dementia (Son and Park, 2022). It is characterized by measurable cognitive decline that does not significantly interfere with daily functioning and thus does not meet the diagnostic criteria for dementia (Lautenschlager et al., 2019; Amrapala et al., 2023; Luo et al., 2024). In particular, amnestic Mild Cognitive Impairment (aMCI), characterized by predominant memory decline, is more strongly associated with AD-related neurodegeneration (Yuan et al., 2022). Consequently, there is growing interest in objective, biomarker-based diagnostic approaches to improve early detection and risk stratification.

In recent years, the ATN framework–based on three core biomarkers: Aβ (A), tau (T), and neurodegeneration (N)–has been widely adopted for the pathological classification of AD. This framework enables the stratification of individuals with Mild Cognitive Impairment (MCI) into distinct subgroups, such as A+T+ (high-risk), A+T− (early pathological stage), and A−T− (low-risk or non-AD) (Kim et al., 2021; Zhang et al., 2021). Although the detection of protein aggregates typically relies on positron emission tomography, this technique remains relatively inaccessible outside specialized centers (Busche and Hyman, 2020; Boccalini et al., 2024). By contrast, structural magnetic resonance imaging (MRI) offers a more widely available, cost-effective, and less invasive alternative (Wang et al., 2024; Zhang J. et al., 2024). In addition to cognitive decline, emotional dysregulation is increasingly recognized as an early feature of preclinical AD and MCI, reflecting dysfunction within limbic circuits (Li et al., 2016; Ten Kate et al., 2017). The amygdala plays a central role in integrating emotional and cognitive processes, and its interactions with the hippocampus are critical for memory modulation (McDonald and Mott, 2017). Through its extensive connections with limbic and cortical regions, the amygdala contributes not only to emotional regulation but also to learning, memory encoding, and the assignment of salience to stimuli (Angrilli et al., 1996; Banks et al., 2007). Disruption of these processes may therefore reflect early involvement of the amygdala and related limbic regions in AD-related neurodegeneration. Accordingly, investigating amygdala subregions may provide valuable insights into the neural basis of cognitive impairment.

Medial temporal lobe atrophy assessed via structural MRI has been established as a key biomarker for the early diagnosis of both AD and MCI (Ten Kate et al., 2017). However, compared with the extensively studied hippocampus, the amygdala–particularly its subregional organization–has received relatively less attention, despite its critical role in both emotional processing and memory modulation through its interactions with hippocampal networks. Previous studies have demonstrated that volume reduction in the amygdala can occur as early as the MCI stage (van der Flier et al., 2004; Jessen et al., 2006; Striepens et al., 2010). More recently, emerging evidence suggests that amygdala subnuclei may exhibit selective and early vulnerability in the AD continuum, highlighting the importance of subregional investigations. However, few investigations have specifically explored amygdala volume alterations across MCI subgroups defined by the ATN classification, particularly at the subnuclear level.

To address this gap, the present study utilized FreeSurfer 7.4.0 to segment amygdala subregions on structural MRI in MCI patients. We aimed to characterize patterns of amygdala atrophy across different ATN-defined MCI subgroups. Additionally, we examined the relationship between these imaging alterations and cognitive performance, using a battery of clinical assessments. This study seeks to provide new insights into the early neurobiological changes in MCI and to identify potential imaging biomarkers for early diagnosis and disease monitoring. We hypothesized that amygdala subnuclei would exhibit differential vulnerability across ATN-defined MCI subgroups, and that these alterations would be associated with cognitive function, providing complementary information to hippocampal atrophy in characterizing early AD-related neurodegeneration.

## 2 Materials and methods

### 2.1 Participants and ATN classification

All data in this study were obtained from the Alzheimer’s Disease Neuroimaging Initiative (ADNI) database^1^. The inclusion of MCI patients were based on the ADNI procedures manual: (1) memory complaint; (2) Abnormal memory function documented by scoring within the education adjusted ranges on the Logical Memory II subscale (Delayed Paragraph Recall, Paragraph A only) from the Wechsler Memory Scale Revised (≤8 for 16 or more years of education; ≤4 for 8–15 years of education; ≤2 for 0–7 years of education); (3) Clinical Dementia Rating (CDR) = 0.5; (4) Mini-Mental State Examination (MMSE) scores between 24 and 30; (5) no dementia and no signal of depression (Geriatric Depression Scale, GDS < 6). The inclusion of HCs was based on the ADNI-2 procedures manual: (1) no memory complaints; (2) normal cognitive performance, MMSE between 24 and 30, and GDS < 6; (3) CDR = 0.

Based on previous studies, this study defined abnormal CSF biomarkers using the following thresholds: Aβ42 concentration < 977 pg/ml was considered A+ and A− otherwise, and phosphorylated tau (p-tau) concentration > 24 pg/ml was considered T+ and T− otherwise (Hansson et al., 2018; Vromen et al., 2023). In alignment with the A/T/N research framework, the A−T+ group was excluded, as it does not lie within the AD pathological continuum. Accordingly, the final analytical sample comprised 54 A−T−, 28 A+T−, and 52 A+T+ MCI participants.

### 2.2 Neuropsychological assessment

To assess cognitive function, group comparisons were conducted using composite episodic memory (EM) and executive function (EF) scores (Gibbons et al., 2012). The composite EM score included performance from the Rey Auditory Verbal Learning Test (RAVLT) and its key indicators–RAVLT-immediate, RAVLT-learning, RAVLT-forgetting, and RAVLT-percent forgetting–as well as the word list learning and recognition components of the Alzheimer’s Disease Assessment Scale-Cognitive Subscale (ADAS-Cog), the word recall task from the Mini-Mental State Examination (MMSE), and Logical Memory I from the Wechsler Memory Scale–Revised. The composite EF score included results from the Digit Symbol Substitution and Digit Span Backward tests, Trail Making Test parts A and B, category fluency tasks (animals and vegetables), the Digit Cancellation Test, and the Clock Drawing Test.

### 2.3 CSF biomarkers

CSF biomarkers were measured using the Roche Elecsys electrochemiluminescence immunoassay (ECLIA) platform, including Aβ42, total tau, and phosphorylated tau (p-tau181), following standardized ADNI protocols.

### 2.4 Ethical approval and informed consent

The ADNI study received ethical approval from the institutional review boards of all participating institutions. Written informed consent was obtained from all participants or their authorized representatives. More details can be found on the ADNI website^2^.

### 2.5 MRI data acquisition and analysis

Magnetic resonance imaging data were obtained from the ADNI database, which employs standardized acquisition protocols across multiple sites and scanners. Detailed acquisition parameters are publicly available through the ADNI repository.

**FIGURE 1:**
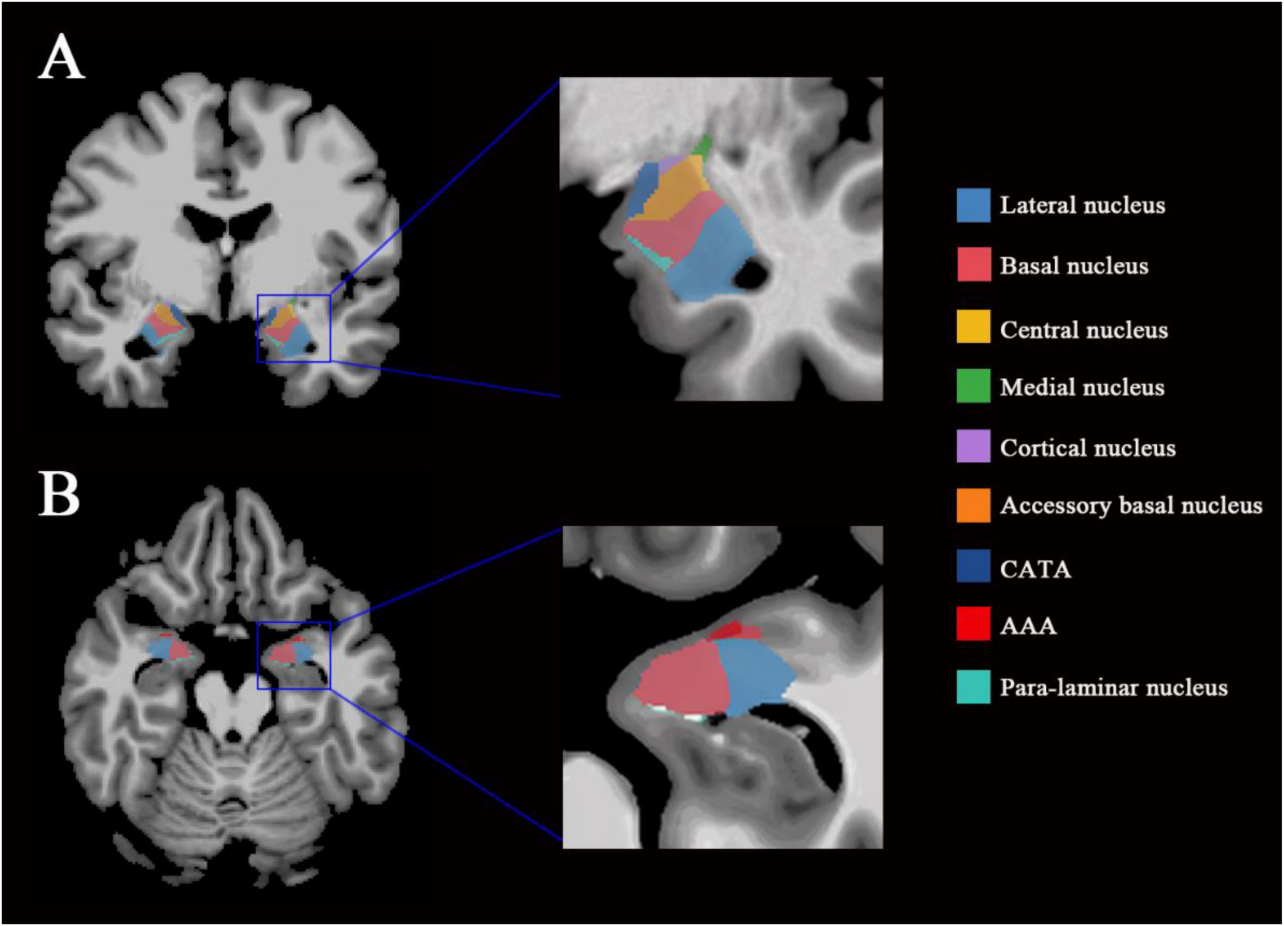
Visualization of amygdala subregions on T1-weighted MRI. **(A)** Representative coronal view showing the bilateral amygdala subregions. **(B)** Representative axial view showing the bilateral amygdala subregions. The boxed regions in the whole-brain images are enlarged to illustrate the spatial distribution of the segmented amygdala nuclei. Different colors indicate distinct amygdala subregions, including the lateral, basal, central, medial, cortical, accessory basal, AAA (Anterior Amygdaloid Area), CATA (Cortical-Amygdaloid Transition Area), and para-laminar nuclei.

T1-weighted images were processed with FreeSurfer 7.4.0 using the “recon-all” command line. This processing includes motion correction and intensity normalization of T1-weighted images, removal of non-brain tissue using a hybrid watershed/surface deformation procedure, automated Talairach transformation, segmentation of the subcortical white matter (WM) and deep GM volumetric structures, and cortical surface reconstruction. As part of this pipeline, images were registered to Talairach space and further aligned to the FreeSurfer fsaverage template to facilitate atlas-based segmentation, with volumetric measures ultimately derived in each subject’s native space. Amygdala subregional segmentation was performed using the “segmentHA_T1.sh” script, which parcellates the amygdala into nine nuclei in each hemisphere, including the accessory basal nucleus, anterior amygdaloid area (AAA), basal nucleus, central nucleus, cortical-amygdaloid transition area (CATA), cortical nucleus, medial nucleus, lateral nucleus, and para-laminar nucleus (Saygin et al., 2017). We visualized the amygdala subregions on T1-weighted MRI, as shown in Figure 1. All segmentation results were visually inspected for quality control, and cases with evident segmentation errors or poor image quality were excluded from further analysis. Finally, subregional volumes were normalized by the estimated total intracranial volume, which is automatically derived by FreeSurfer based on atlas scaling during Talairach registration and is widely used for head-size correction. Although this standardized pipeline improves comparability across subjects, potential variability related to anatomical differences and registration cannot be entirely excluded.

### 2.6 Statistics

Analysis of variance and Bonferroni *post-hoc* tests were used to evaluate group differences regarding demographic, neuropsychological, and clinical data. The categorical variables were analyzed using chi-square tests.

For the MRI measures, a MANCOVA, followed by *post hoc* comparison, was applied to test the differences among groups. Age, sex, and education were included as covariates based on their known influence on brain structure and cognitive performance, even in the absence of significant group differences. Spearman’s correlations were conducted to examine possible relationships between the volume of atrophic subfields and the neuropsychological outcomes controlling for age, sex, and education to account for potential confounding effects. Multiple comparisons were corrected using the false discovery rate (FDR) with the Benjamini–Hochberg procedure (q < 0.05).

## 3 Results

### 3.1 Demographic and clinical features

Differences in demographic characteristics, cognitive assessments, and CSF biomarkers among MCI subgroups are presented in Table 1. Significant age differences were observed among the three MCI subgroups, with the A+T+ group being the oldest and the A−T− group the youngest; no significant differences were found in sex distribution or years of education.

**TABLE 1:** Group comparisons of demographic characteristics, clinical scale scores, and CSF biomarkers.

General factors	A−T−	A+T−	A+T+	F-values (χ^2^)	*P*-values
Age (years)	68.97 (7.65)	71.68 (7.30)	73.16 (5.79)	4.982	0.008^a^
Sex (Male/Female)	20/34	13/15	20/32	0.723	0.697
Years of education	15.91 (2.64)	16.46 (2.57)	16.33 (2.65)	0.534	0.588
MMSE	28.06 (1.89)	28.18 (1.74)	27.38 (2.18)	2.100	0.127
MoCA	23.79 (3.06)	23.04 (2.52)	22.76 (3.61)	1.402	0.250
RAVLT-immediate	38.43 (9.42)	34.68 (9.21)	33.10 (8.94)	4.620	0.012^a^
RAVLT-learning	4.91 (2.24)	4.36 (2.09)	3.73 (2.39)	3.559	0.031^a^
RAVLT-forgetting	4.43 (3.93)	4.57 (2.17)	5.10 (2.51)	0.654	0.522
RAVLT-prec-forgetting	46.08 (56.25)	57.15 (29.42)	65.50 (30.51)	2.757	0.067
EM	0.49 (5.20)	0.30 (1.05)	0.05 (0.65)	5.113	0.007^a^
EF	0.65 (0.91)	0.23 (1.05)	0.24 (0.84)	3.310	0.040^a,b^
Aβ_42_	1484.25 (255.03)	726.19 (199.64)	622.09 (159.11)	249.840	<0.001^a,b^
t-tau	201.89 (44.35)	178.88 (46.61)	383.71 (130.72)	73.112	<0.001^a,c^
p-tau	17.36 (3.98)	16.30 (4.68)	40.58 (16.54)	75.415	<0.001^a,c^

Values are presented as mean (standard deviation, SD), unless otherwise specified. MMSE, Mini-Mental State Examination; MoCA, Montreal Cognitive Assessment; RAVLT, Rey Auditory Verbal Learning Test; EM, episodic memory; EF, executive function; Aβ, Amyloid-beta protein; p-tau, phosphorylated tau protein; t-tau, total tau protein; A+T+, abnormal Aβ42 and abnormal p-tau; A+T−, abnormal Aβ42 and normal p-tau; A−T−, normal Aβ42 and normal p-tau. ^a^Comparison between A−T− and A+T+; ^b^Comparison between A−T− and A+T−; ^c^Comparison between A+T− and A+T+.

In terms of cognitive assessments, although MMSE and MoCA scores did not significantly differ among the subgroups, the A+T+ group showed poorer performance across multiple specific cognitive domains. For example, RAVLT-immediate and RAVLT-learning scores were significantly lower in the A+T+ group compared to the A−T− group. EM scores were also lowest in the A+T+ group, with a significant difference compared to the A−T− group. On EF tests, both the A+T− and A+T+ groups scored lower than the A−T− group.

### 3.2 MRI volume

**FIGURE 2:**
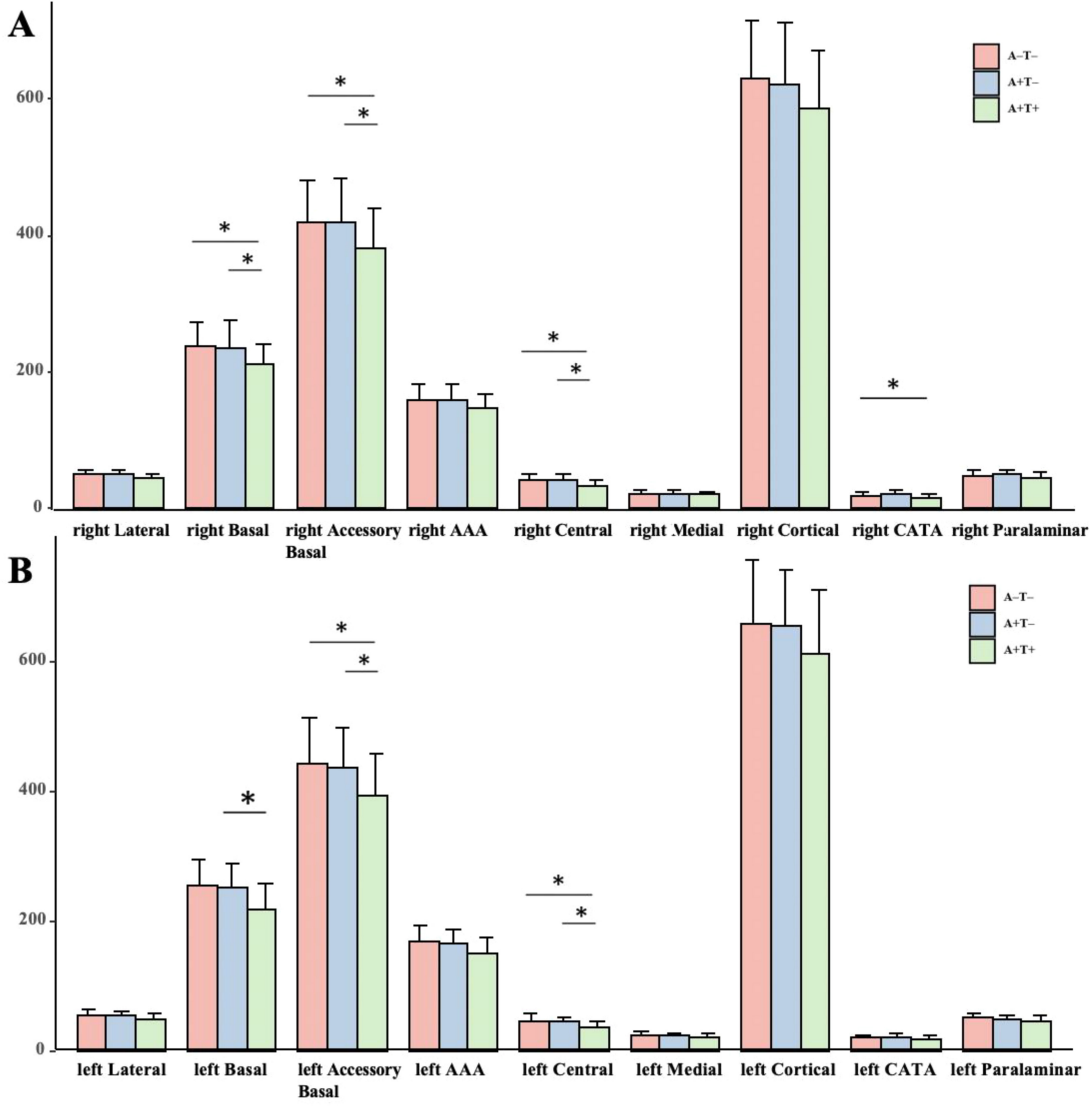
Grouped bar chart with two panels **(A,B)**, each comparing amygdala subregional volumes across three groups: A−T− (red), A+T− (blue), and A+T+ (green). The Y-axis displays volume values, and the X-axis lists nine amygdala subregions: lateral, basal, accessory basal, AAA (Anterior Amygdaloid Area), central, medial, cortical, CATA (Cortical-Amygdaloid Transition Area), and para-laminar nuclei. *p < 0.05 after FDR correction. **(A)** Displays right hemisphere subregional volumes, while **(B)** displays left hemisphere subregional volumes. A+T+: abnormal Aβ42 and abnormal p-tau; A+T−: abnormal Aβ42 and normal p-tau; A−T−: normal Aβ42 and normal p-tau.

As shown in Figure 2 and Table 2, the A+T+ group exhibited varying degrees of atrophy in the amygdala and its subregions compared to the A−T− and A+T− groups, including right basal nucleus, right accessory basal nucleus, right central nucleus, left accessory basal nucleus, and left central nucleus. The A+T+ group showed significant atrophy in right CATA compared to the A−T− group, and in left basal nucleus compared to the A+T− group. No significant differences were observed between the A+T− and A−T− groups.

**TABLE 2:** Amygdala volumetric measures among ATN groups.

Volume nucleus	A−T− (Mean ± SD)	A+T− (Mean ± SD)	A+T+ (Mean ± SD)	*F*-value	FDR-p
r Lateral	658.31 ± 98.42	655.16 ± 84.57	611.42 ± 98.72	4.103	0.0306
r Basal	442.79 ± 69.88	437.05 ± 62.11	393.45 ± 63.98	7.77	0.0033^a,c^
r Accessory_Basal	254.77 ± 40.26	250.92 ± 38.14	218.86 ± 39.79	10.43	<0.001^a,c^
r AAA	53.95 ± 8.92	53.39 ± 8.47	48.91 ± 7.68	3.908	0.0345
r Central	46.12 ± 11.16	45.30 ± 7.83	37.05 ± 8.70	11.201	<0.001^a,c^
r Medial	20.60 ± 4.24	20.68 ± 6.40	18.07 ± 4.83	3.426	0.045
r Cortical	24.57 ± 4.31	24.26 ± 4.02	21.74 ± 4.32	4.897	0.0171
r CATA	168.98 ± 24.21	165.02 ± 21.05	149.75 ± 25.21	7.298	0.0033^c^
r Paralaminar	50.33 ± 8.25	49.96 ± 6.47	46.28 ± 7.52	4.708	0.0185
r Whole_amygdala	1720.42 ± 249.02	1701.75 ± 223.80	1545.53 ± 243.70	7.517	0.0033^a,c^
l Lateral	629.93 ± 85.45	622.39 ± 89.92	587.76 ± 84.05	3.282	0.0482
l Basal	420.47 ± 59.24	419.94 ± 63.24	381.96 ± 56.81	5.832	0.0095^c^
l Accessory_Basal	238.04 ± 35.37	235.71 ± 41.69	210.34 ± 31.85	6.726	0.0054^a,c^
l_AAA	49.94 ± 7.10	49.66 ± 7.68	45.64 ± 6.50	4.437	0.0225
l Central	42.13 ± 8.10	41.30 ± 9.07	34.54 ± 8.29	9.414	<0.001^a,c^
l Medial	18.36 ± 4.68	20.24 ± 8.36	16.49 ± 4.49	3.561	0.0415
l Cortical	22.08 ± 4.15	21.75 ± 5.10	20.28 ± 3.62	1.148	0.3371
l CATA	159.83 ± 22.47	158.83 ± 24.24	146.06 ± 23.08	3.729	0.0378
l Paralaminar	48.54 ± 7.74	49.58 ± 7.16	45.73 ± 7.24	3.32	0.0477
l Whole_amygdala	1629.32 ± 210.64	1619.40 ± 232.90	1488.80 ± 205.77	5.659	0.0095

AAA, Anterior Amygdaloid Area; CATA, Cortical-Amygdaloid Transition Area; SD, standard deviation; A+T+, abnormal Aβ42 and abnormal p-tau; A+T−, abnormal Aβ42 and normal p-tau; A−T−, normal Aβ42 and normal p-tau. ^a^Comparison between A−T− and A+T+; ^b^Comparison between A−T− and A+T− (no significant differences observed); ^c^Comparison between A+T− and A+T+. l, left; r, right.

### 3.3 Correlation analysis of significant brain differences and neuropsychological tests

**FIGURE 3:**
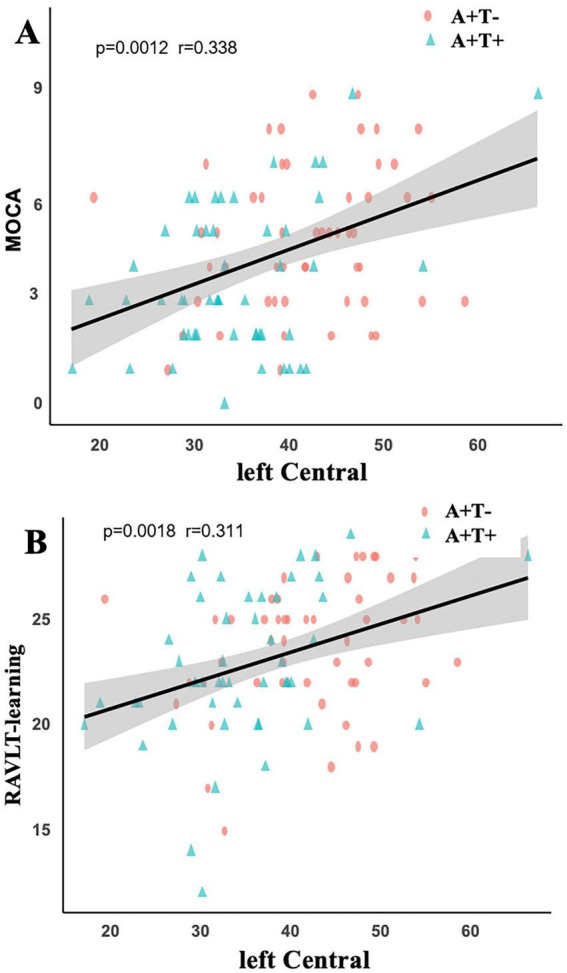
**(A)** Significant positive association between left central amygdala volume and MoCA scores. **(B)** Significant positive association between left central amygdala volume and RAVLT-learning scores. A+T+, abnormal Aβ42 and abnormal p-tau; A+T–, abnormal Aβ42 and normal p-tau.

As shown in Figure 3, correlation analysis revealed a positive association among A+T− and A+T+ groups between the left central amygdala and both MoCA (*p* = 0.0012, *r* = 0.338) and RAVLT-learning (*p* = 0.0018, *r* = 0.311) after Bonferroni correction.

## 4 Discussion

The primary objective of this study was to identify morphological changes in specific amygdala subregions across different ATN subgroups. Although subcortical atrophy of the amygdala was observed across all groups, no significant differences were found between the A+T− and A−T− groups. The affected subregions included bilateral basal nucleus, bilateral accessory basal nucleus, bilateral central nucleus, and right CATA. As expected, atrophy in some of these subregions was significantly associated with cognitive performance.

In line with previous research, our study found that amygdala atrophy progressively worsens as the AD advances (Qu et al., 2023). Compared with both A−T− and A+T− groups, the A+T+ group exhibited significant atrophy in the bilateral accessory basal, bilateral central, and right basal subregions of the amygdala. Previous studies have reported neurofibrillary changes in the amygdala, including the central nucleus, during early stages of AD, which may reflect the vulnerability of these regions to AD-related pathological processes (Kromer Vogt et al., 1990; Braak and Braak, 1995). The central nucleus is involved in integrating emotional and autonomic responses through its connections with the hypothalamus, basal forebrain, and brainstem (Yang et al., 2023; Xiang et al., 2025). Importantly, beyond its traditional role in emotional processing, the amygdala also contributes to memory-related processes through its interactions with the hippocampus and broader limbic networks (Li et al., 2016; McDonald and Mott, 2017). Our correlation analyses showed that central nucleus volume in A+T+ individuals was associated with RAVLT-learning and MoCA scores. This pattern supports the notion that specific amygdala subnuclei may contribute to cognitive impairment through their integration within limbic memory-related circuits rather than through isolated structural changes. Taken together, these findings suggest that amygdala subregional alterations may reflect early dysfunction at the systems level, linking structural changes to both cognitive and affective domains.

Additionally, other amygdala subnuclei–including the basal and accessory basal nuclei– also exhibited atrophy in the A+T+ group. Previous studies have also reported neuronal loss and pathological burden in multiple amygdala subregions in AD (Kromer Vogt et al., 1990; Salman et al., 2024). Given its role in updating stimulus–value associations via connections with the orbitofrontal cortex, the basal nucleus is essential for emotional and social cognition (Alheid, 2003; Scheef et al., 2012; Epelbaum et al., 2017). Therefore, atrophy in these subregions may contribute not only to cognitive impairment but also to broader alterations in social and affective functioning observed in early AD and MCI. In addition, the A+T+ group showed significant atrophy in the right CATA region compared to the A−T− group. The CATA is characterized by its dense cholinergic and dopaminergic innervation, distinguishing it from adjacent regions like the piriform cortex and other amygdala subregions, and by its direct projections from both the main and accessory olfactory bulbs (Bzdok et al., 2013; Bell et al., 2022). Due to its unique anatomical location and functional roles, damage to the CATA may contribute to cognitive impairments observed in patients (Canteras et al., 1995; Cádiz-Moretti et al., 2016).

Furthermore, most atrophy was observed in the right amygdala subregions. Anderson (2017) reported that patients with right amygdala damage exhibited impairments in recognizing emotional expressions, whereas patients with left-sided lesions showed no significant difference from healthy controls. Similarly, a case study by Wang et al. (2002) found that a patient with right amygdala and bilateral anterior cingulate damage following stereotactic brain surgery demonstrated a marked deficit in recognizing fearful faces. Taken together with our findings, these studies suggest a possible rightward predominance, that the right amygdala may play a more critical role in the disruption of recognition abilities (Lieberman et al., 2007; Guadagno et al., 2020; Chen et al., 2023). Future studies incorporating formal laterality analyses are needed to validate potential hemispheric differences.

Several limitations should be acknowledged in this study. First, significant age differences among groups may introduce potential confounding effects. Although age, sex, and education were statistically controlled in all analyses, residual confounding cannot be entirely excluded, particularly in a cross-sectional design. Moreover, this age gradient may reflect the natural progression of AD pathology across the ATN continuum. Second, the cross-sectional design restricts our ability to infer causal relationships or track longitudinal changes in amygdala subregional atrophy. Thirdly, although FreeSurfer provides a widely used and reproducible framework for automated amygdala subnuclear segmentation, its accuracy may be limited for small nuclei such as the central amygdala. Therefore, the present subregional findings should be interpreted with caution and validated in future studies using higher-resolution imaging or complementary segmentation approaches. Finally, while we included cognitive measures, other potentially relevant behavioral or neuropsychiatric symptoms were not assessed and may provide additional insights in future research.

In summary, this study reveals that specific amygdala subregions, particularly the central, basal, accessory basal, and CATA nuclei, exhibit progressive atrophy across the ATN spectrum. The central nuclei alterations are significantly associated with cognitive decline, suggesting their potential involvement in AD-related pathological processes. Notably, our findings highlight the potential vulnerability of the right amygdala, reinforcing its critical involvement in emotion recognition and social cognition. These results, consistent with both neuroimaging and neuropathological studies, provide further evidence for the role of amygdala subregional degeneration in the pathophysiology of AD.

## Data Availability

Publicly available datasets were analyzed in this study. This data can be found here: The data for this study are sourced from the public database ADNI, and the link is as follows: https://adni.loni.usc.edu/.

## Footnotes

1
http://adni.loni.usc.edu

2
www.adni-info.org
